# Supplementary material for: Nitrate Modulates Lateral Root Formation by Regulating the Auxin Response and Transport in Rice
Source: Genes (Basel). 2021 Jun 1;12(6):850. doi: 10.3390/genes12060850 (PMC8229813; doi:10.3390/genes12060850)
Supplement: Supplementary file 1 [file genes-12-00850-s001.zip › Supplemental data-Manuscript with track.pdf]

Supplementary Table S1 The primers for qRT-PCR of *ARF1* and *PIN2* genes

Gene	Primer sequence
OsARF1	5'-ATCGCTATATGGAATCACTGA-3'
	5'-GAGCCTCTTCACCTTCAA-3'
OsPIN2	5'-CAACACCTACTCCAGCCTC-3'
	5'-TGGACCAGTCAAGAACCTC-3'
OsActin	5'-CAACACCCCTGCTATGTACG-3'
	5'-CATCACCAGAGTCCAACACAA-3'

A

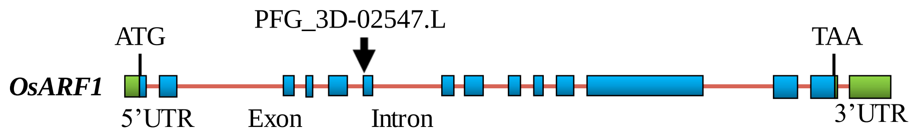

B

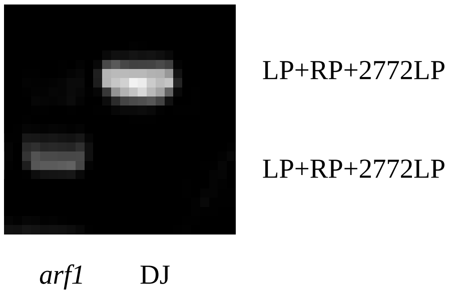

Supplementary Figure S1 Identification of the T-DNA insertion *arf1* mutant. (A) Gene structure of *ARF1* and the T-DNA insertion site. The exons and introns are indicated by bold boxes and lines, respectively. Insertion site of T-DNA in the mutant line is indicated by arrowhead showing the name of mutant. (B) Identification of homozygous T-DNA insertion mutant by two rounds of PCR.

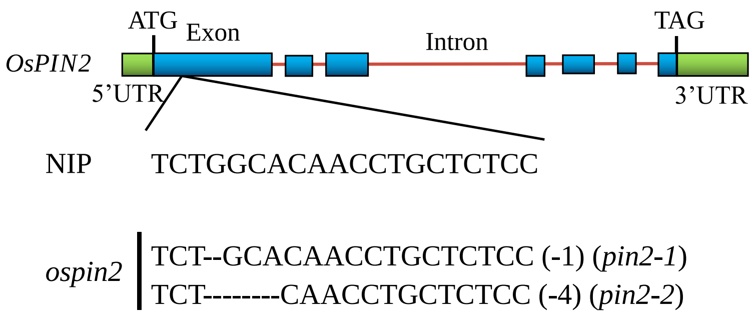

Supplementary Figure S2 Sequencing verification of CRISPR-edited *PIN2* knockout mutants. Seedlings were grown in hydroponic medium containing normal nutrition.
